# Supplementary material for: Edible Flowers of Tagetes erecta L. as Functional Ingredients: Phenolic Composition, Antioxidant and Protective Effects on Caenorhabditis elegans
Source: Nutrients. 2018 Dec 18;10(12):2002. doi: 10.3390/nu10122002 (PMC6316237; doi:10.3390/nu10122002)
Supplement: Supplementary file 1 [file nutrients-10-02002-s001.pdf]

Table S1. Effects of orange cultivar of *T. erecta* flowers extracts on the viability of wild-type *C. elegans*. The results are presented as mean of % viability ±SEM. Not significance differences between treatment and control groups were found ($p > 0.05$).

Concentration (µg/mL)	Orange Cultivar	*p*-Value
Control	90 ±2	
2000	91 ± 2	>0.05
1000	89 ± 3	>0.05
750	91 ± 2	>0.05
500	96 ± 2	>0.05
250	93 ± 1	>0.05
100	92 ± 2	>0.05
50	89 ± 2	>0.05

Table S2. Effects of yellow variety of *T. erecta* flowers extracts on the viability of wild-type *C. elegans*. The results are presented as mean of % viability ±SEM. Not significance differences between treatment and control groups were found ($p > 0.05$).

Concentration (µg/mL)	Yellow Cultivar	*p*-value
Control	93 ± 2	
2000	96 ± 2	>0.05
1000	96 ± 2	>0.05
750	96 ± 2	>0.05
500	93 ± 3	>0.05
250	90 ± 3	>0.05
100	90 ± 2	>0.05
50	91 ± 2	>0.05
